# Is the Young Man Safe?—A Talk to Fathers and Young Men

**Published:** 1913-09

**Authors:** Theo. Y. Hull

**Affiliations:** Secretary Texas Society of Social Hygiene and Eugenics


### Is The Young Man Safe?—A Talk to Fathers and Young Men.

BY THEO. Y. HULL, M. D., SECRETARY TEXAS SOCIETY OF SOCIAL HYGIENE AND EUGENICS.

When the messengers bearing news of the defeat of Absalom came to David, the first thought of the King was expressed in the question: "Is the young man safe?" When they answered that he had fallen a victim to his own rebellion, the father, though king he was, cried in anguish, "Would God I had died for thee." The question of David is and always will be the question asked by the anxious parent. The cry of anguish that sprang from David's lips is still the cry of the broken heart.

But it is not my purpose to read you a lecture on the wayward or prodigal son. Rather it is my purpose to point out the evils of a certain nature that beset the young man before he has attained the so-called "years of discretion," while he is still amenable to your teaching, and also to show the lasting consequences that flow from such evil indulgence.

In the discussion of this subject I assume that you understand that in the scheme of nature the highest function in the animal life lies in the reproduction of its kind. In a biological sense this is the most important function of the body. The sexual instinct is a part of that universal plan which provides for the perpetuation of the species. It is easy to see how this function, so important to life itself, could be degraded, perverted, and made inimical to the perpetuation of the race by abuse. To regulate and safeguard the propagation of the species, marriage became an institution of human society legalized by the State and sanctioned by the Church. The social aim of marriage is the creation of the family—the raising of children. But the social aspect of the case is not satisfied by the mere production of children. The welfare of society demands that they be born under conditions that insure physical and mental health. The mere propagation of the species is not enough. It is a crime against society itself to bring into existence physical and mental wrecks.

In this discussion I shall also assume that what seems to be, both on the part of the parent and society, *rank indifference* to the future welfare of the young man and the young woman—what is sometimes designated as the conspiracy of silence—is due largely, if not entirely, to ignorance and false modesty—the latter in a sense being a product of the former—the lack of proper instruction. Many of the great crimes of the past have been due to ignorance, and some crimes against public morality are being perpetrated even now by the same cause, but only in regard to the sex-life of the people can it be said that modesty, the crowning virtue of womanhood and manhood, becames a source of great danger, a positive evil, and then only when perverted by false education.

It is the object of the social hygiene movement to arouse a desire for proper information and to place at the disposal of every intelligent parent, every young man and young woman of proper age, such information as may be helpful in safeguarding the future of the individual lives in their charge. Social hygiene means clean living. It is particularly the young man and the young woman to whom this information may be the most useful. Those who, in a few years, expect to assume the duties of manhood and womanhood in the home, in the family, in the country, it hopes to teach.

It is only natural to suppose that parents are interested in the future of their children. It is only natural that young people about to enter upon the real duties of life should be interested in what that life may be to them. Therefore it is my purpose in this talk to point out to you, who are fathers, and to you, who hope to be fathers, those facts in regard to this social evil, often spoken of as the venereal peril, that may be helpful to you in safeguarding parenthood, that it may prove the crowning blessing, rather than the uncrowning curse, of your life. No man lives unto himself, though the selfish sometimes imagine that they do. No truth has been more frequently verified than the statement in Numbers 14-18, "and by no means clearing the guilty, visiting the iniquity of the fathers upon the children unto the third and fourth generation."

It is one of the functions of the human mind to inquire into the secrets of nature. There are many things about his own life that are more or less mysterious to the youth. It is a mistake to withhold proper information and thereby enshroud these things in greater mystery. Do not imagine that because you have grown up, and uninstructed in these matters, have escaped the more serious pitfalls of ignorance, that your son, under different circumstances, will be equally safe. Do not imagine that, because you have joined in the conspiracy of silence, others will be equally silent. The information, or rather misinformation, will be had, and it will come, in many instances, through the channels of his chance associates, vulgar stories, or from fakers whose glittering advertisements adorn the pages of nearly every newspaper. He will be led by these to believe that certain conditions that every youth observes are indications of lost manhood, and what not. The information received through these channels tends to degrade womankind in his view. The high ideals of woman so carefully instilled by the mother are replaced by lower ideals. Many a promising youth dates his downfall from this moment. Remember that his standard of life will be no higher that his ideals of womanhood. In fact, the state of society rises and falls with its estimate of woman.

Is there danger to the young man? Irregular and clandestine sexual relations are always dangerous. This fact should be impressed indelibly in the mind of the young man. The reports of the various vice commissions show that such relations are astoundingly frequent. There is no need here to discuss the causes of prostitution. The central cause is the unchastity of man. The prostitute is merely the purveyor of infection from man to man. She is primarily in most cases the victim of his carnal desires. But this fact does not make the evil of prostitution any less terrible, some writers assert that there are over one-half million public prostitutes in the United States. They also state that there are three times as many clandestine prostitutes. If one-third of these are really engaged in this nefarious traffic, it indicates an amazing degree of slavery to vice in this land, proclaimed in song as the "Land of the free and home of the brave." Among these the young man has "his fling." Each one is a probable source of infection. It is estimated that 50 per cent. of the nearly 15,000,000 boys in the United States will be infected before they reach their majority. I repeat, is there danger to the young man?

I do not need define what I mean by the "venereal peril." More or less vaguely you comprehend its significance. You understand, because you have been educated up to it, that smallpox, cholera and yellow fever are something to flee from. You are beginning to realize that tuberculosis is a real peril of great magnitude; but you have hardly made up your mind, because this instruction has been omitted, that the venereal diseases are a menace of equal seriousness to your happiness.

The two diseases that constitute the chief factors in this venereal peril I wish to discuss with you. Suppose, for the sake of illustration, that your son—not your neighbor's son—through ignorance of the highest functions of his body—ignorance for which you are largely responsible—or through misinformation gratuitously received, contracts one of these diseases. What is the penalty that he may pay for such improper indulgence? As I said before, no man lives unto himself. The penalty includes, or may include, others. It has a fourfold aspect. The consequences that may follow, and probably will follow, are (1) those that affect himself, (2) those which, if he marries, fall upon an innocent and unsuspecting woman, (3) those which are manifest in the offspring and (4) those which the State must bear.

Let us consider first what is commonly considered, by the laity at least, the more serious of these diseases—syphilis. Its origin is shrouded by the mists of the ages. It probably was inherited from the barbaric times. Human bones taken from the excavations of Ancient Egypt and Babylon give every indication of syphilitic changes. In the Bible may be found undoubted reference to it. "James IV (1497) ordered all persons suffering from syphilis to leave Edinburgh on pain of being branded on the cheek." We are not dealing with a new condition, but an old condition, about which society, until very recently deemed it wisest to remain very silent.

Since the man is the chief sinner through his promiscuous indulgences, let us consider the untoward effects upon him first. It has been stated by what seems good authority that not less than 10 per cent. of the adult male population has at one time or another had syphilis. Morrow says that "the prevalence of syphilis is variously estimated at from 5 per cent to 18 per cent." While this statement of the frequency of infection seems almost incredible, it is, nevertheless, according to the experiences of physicians treating chronic diseases, not far from the truth. Syphilis is not a transient complaint. Its serious consequences may become manifest ten, twenty, or thirty years after the infection. Omitting from consideration the acute effects of syphilis,—the so-called benign effects—such as inflammatory changes in the eye (iritis), the ear (deafness), the kidneys (nephritis), the liver (icterus) and malignant syphilis, the graver chronic effects fall upon important organs of the body. Among a series of 4400 cases mentioned by Fournier (4000 men and 400 women) such changes had occurred in about the following proportion: Syphilides (skin eruptions resulting from syphilis), 1451; Tabes (locomotor ataxia 631) and other changes in the spinal cord (135), 766; brain, 758; bones, 519; eye, 110; kidney, 31; lungs, 23; heart and aorta, 19, and liver 9. The "brand upon the cheek" ordered by King James for all syphilitics who would not leave Edinburgh would be scarcely more noticeable than the cutaneous syphilides so frequently found in tertiary syphilis. But the nervous system becomes the chief victim of chronic syphilis. Not only paralysis (motor), mental decay (insanity), and often death follow brain lesions, but locomotor-ataxia and other nervous disturbances follow lesions of the spinal cord.

The mortality statistics for 1910 indicate that over 2400 adults died from locomotor-ataxia in the United States during that year. Apoplexy, a large per cent of which is due to the effects of syphilis upon the arteries, in the same year ended the careers of about 60,-000, while general paralysis of the insane and softening of the brain brought to an untimely end 6000 more. Fournier says that of 354 cases of brain syphilis, the termination of which he knew, 22 per cent were cured, 19 per cent died, 59 per cent survived with permanent infirmities, such as paralysis and mental decay, almost equivalent to death. Additional cases might be given almost indefinitely, but these should be sufficient to prove the terrible penalty man pays for his evil indulgences.

Let us now consider the effect of syphilis upon the woman. Thousands upon thousands of syphilitic men marry every year. An innocent woman with the sublimest faith and a trust worthy of the highest reward, joins hands with and intrusts her future to one of these. We might forgive the man if the consequences of his improper indulgence ended with himself; but when these same consequences fall upon one whose faith in his manhood cannot be described in words, his act becomes a crime of the darkest nature, though unnamed by law, and merits the utter condemnation of every bit of manhood in the land. The syphilitic who marries is almost certain to infect his wife. Just how frequently syphilitic infection occurs in married life is difficult to determine. Fournier (Paris) states that twenty per cent of all syphilis in women is contracted from the husband. Morrow states that in his practice (New York) seventy per cent of women with this disease were married, and had contracted it from their husbands. But, once contracted, no matter how, it runs about the same course in woman that it does in man, with the additional penalty in woman that she forfeits "her highest destiny in being created a woman." The instinct of maternity is present in every normal woman. Morrow says, "It is only in the confessional of the consulting room that one learns of the intense, insistent craving on the part of many women for children, and of the wretchedness and disappointment they suffer in being condemned to pass their existence in a childless wedlock." From the disease a few recover, about as many die, but the larger proportion live on in varying degrees of physical and mental incapacity.

The census report for 1910 gives for the registration area over 3200 deaths from syphilis. Over one-half of these were infants under one year. This number represents those in which the direct cause of death is admitted to be syphilis. While syphilitic infection in man is followed by terrible consequences, and the same infection in woman by the same and other consequences, the most disastrous effects fall upon the offspring. The disease in this class of cases is often spoken of as hereditary syphilis. Morrow says that "no other disease is so susceptible of hereditary transmission, so pronounced in its influence,, and so fatal to the offspring." There are probably very few people who realize the terrible influence of chronic disease on infant mortality and morbidity. When nearly twenty per cent of all infants born die during the first year of their existence, such a condition constitutes very nearly a national calamity. Just how much of this mortality is due to any one specific cause no one can estimate, but, directly or indirectly, syphilis is a potent factor. Fournier states that in 200 marriages where the man had latent syphilis and the woman remained uninfected (?) 403 pregnancies occurred. Of these 288 resulted in living children, and 115 resulted in abortions, stillbirths and infants dying in a few days after birth; 28 per cent died as a result of the paternal latent infection. In fourteen marriages of syphilitic men (active), 45 pregnancies occurred. There resulted 8 living children, 6 of whom were syphilitic; 29 abortions, stillbirth, etc., and 8 died soon after birth. There were 37 deaths in this group, making a mortality of 82 per cent. In the case of 100 married syphilitic women, there were 208 pregnancies. Of these 60 living children were born, and 148 resulted in abortion, stillbirths or infants dying soon after birth. This gives an infant mortality of 71 per cent in maternal syphilis. This occurred in private practice. In hospital practice the destruction of the offspring rises to 86 per cent. In 1500 cases occurring in syphilitic families, the average death rate among the offspring was 68 per cent. Terrible as this may seem, death is not the only result. In many cases death is even to be preferred. Suppose these children do live, it is not infrequently that they show some of the various stigmata of degeneration. These may manifest themselves in both physical and mental defects. The low vitality (infantile senility or fragility of life) of such offspring is shown in the frequency with which they contract and succumb to the minor diseases which healthy children either escape altogether or pass through without injury. Cleft-palate, club-feet,, or pelvic deformity may be the mark of the disease. Hydrocephalus, idiocy, and various degrees of mental and moral delinquency may appear.

Neither society nor the State escapes paying a high penalty for its blindness and seeming unwillingness to treat the venereal peril openly. It is said that 10 to 12½ per cent of all sickness directly and indirectly may be traced to syphilis. What per cent of the patrons of charity organizations, what per cent of the inmates of our poor-houses, and what per cent of the insane are victims of syphilis? What is the public expense for the maintenance of institutions for the care of such?

The second factor in the venereal peril to which I wish to call your attention is gonorrhoea. It is a contagious disease due to another micro-organism, and is contracted under much the same condition of promiscuous sexual relations. Its origin, also in all probability like the origin of many other infectious diseases, is concealed in the mist that enshrouds the dawn of civilization. Today it is the most wide-spread of all the contagious diseases. Like syphilis, it has no respect for social position and ramifies through every class of society. Most of our actual knowledge of this disease dates from the discovery of the gonococcus in 1879 by Neisser. The result of modern studies shows it to be a much more serious disease than commonly thought to be, even by the profession itself.

Suppose now again, for the sake of the discussion, that your son, escaping the graver infection, syphilis, contracts gonorrhoea. Where did he get it? From a prostitute. Morrow says that 75 per cent of the adult male population have had this disease at one time or another. Noeggerath asserts that 800 in every 1000 married men in New York have or have had gonorrhoea. Some contract the disease as early as the fifteenth year. In 1000 cases, 122 occurred in boys under twenty. At least 10 per cent is said to be contracted during the academic age. Among the poorer classes 13 per cent contract it before the twentieth year. From this grave condition serious consequences must flow. These fall upon the man, the woman and the child. An old aphorism says, "We know when a gonorrhoea begins, but we know neither when nor how it will finish." It is not the simple harmless disease, even in man, it was once thought to be. Its systemic effects may compromise his health and even endanger his life. From a local urethritis, the inflammation may extend to the bladder and kidneys, with serious consequences. Even blindness and rheumatism may be the price exacted. Sterility, another result, is much more frequent in men than is commonly supposed. Kehren found in 96 childless marriages, the husband was sterile in 30 per cent of the cases. It is said by some authorities that in one-half the sterile marriages the cause may be traced to the husband's gonorrhoea.

Suppose this young man, thinking himself cured, marries. Noeggerath points out that 90 per cent of men with gonorrhoea marry uncured. What penalty must the woman pay for her blind faith in man? It is a sad tale of woe. Less terrible woes have been the cause of armed conflicts.

The effects of gonorrhoea on the woman have not until recently received any serious study. Even from the beginning, the infection is a most serious matter. From its primary seat in the cervical mucosa, it may spread to the endometrium, later invading the Fallopian tubes, the ovaries and the peritoneum. With this begins the long train of woes to which the woman may be subject,—ill health, sterility, pelvic inflammation preceding surgical operations, and sometimes even death. Each menstrual epoch becomes a period of danger of spreading infection, and pregnancy a danger even to the gonorrhoeal woman's life.

Under syphilis we discussed the frequency of abortion in syphilitic woman. Gonorrhoea is also a potent factor in that most disastrous accident to the pregnant woman. Noeggerath found that 19 out of 53 gonorrhoeal women that became pregnant aborted. Fruninsholz reports 101 such pregnancies—71 went to the full term, 23 aborted, and 7 were born prematurely. Saenger considers gonorrhoea equal to syphilis in producing abortion.

At the present moment pelvic inflammations are the curse of young womanhood. Within a few years an appreciable per cent of young married women find their way into the hospital, many undoubtedly innocent of infection, but a much larger number due to infection.

In 1901 the American Medical Association appointed a special committee to study and report upon the cause of pelvic inflammations in women. That report should be interesting reading to the father whose daughter is about to marry. A large number of letters were sent out to specialists in diseases of women, both in this country and in Europe. The average of all reports received indicated that gonorrhoeal infection was the cause of 47 per cent of all cases. Competent authorities regard this as too low. Peterson says the more the disease is studied, the higher the percentage. Pozzi and Frederic, men of large experience, gave 75 per cent due to gonorrhoea. Humiston claimed 90 per cent and Price, with a record of 1000 abdominal sections, attributed 95 per cent as directly due to gonorrhoea.

Since the most important function of the body lies in the reproduction of its kind, gonorrhoea, by inducing sterility in woman strikes a serious blow at the highest function in nature, and deprives her of the one great object of her life. Census reports in Europe indicate that one marriage in every eleven is sterile. In this country of 16,000,000 marriages, one in every seven is reported sterile, and in some parts of the country the number increases to one in every four or five. Of course, a sterile marriage may be the fault of either the man or the woman, but in either event the cause is the same. Neisser declares that gonorrhoea causes 45 per cent of the sterility in woman. Noeggerath says 50 per cent are due to this cause, and reports 81 women with the disease, 49 of whom were sterile, and the other 32 bore only 39 children. Leir-Ascher reports 227 women, of whom 121 were sterile because of gonorrhoeal infection. Other writers give even higher percentages. If we believe the social aim of marriage to be the creation of the family, and the family to consist of the parents and children, or even that children are a desirable outcome of marriage, these statements of sterility in both men and women should cause you to think.

But there is another effect that falls not upon the man or woman but upon the still more helpless infant. Our sense of chivalry ascribes to a pure woman the highest nobility. A healthy infant should be in our eyes the most beautiful thing in the world, and the most to be desired. Our experience shows that neither beauty nor purity escapes, and while this disease cripples the man, and maims the woman, it puts out the eyes of the infant and even sometimes destroys its life.

The frequency of abortion and premature births due to gonorrhoeal infection I have already referred to. It is said that from 10 per cent to 30 per cent of all blindness is due to this infection. The report of the Committee of Seven gave 1941 cases of gonorrhoea in women, 265 children of whom had purulent ophthalmia, which meant more or less permanent blindness. Neisser reports 30,000 cases of blindness in Germany due to this cause.

Over some of these horrid details I would like to drop a curtain, but silence, and hiding the truth rarely correct an evil. But when an evil strikes the absolutely helpless, it should arouse in us the strongest desire to correct it. It is estimated that one child in every 200 born in New York State loses its sight from gonorrhoeal infection. Of the 6200 totally blind in the same state, 32 per cent are sightless from this disease. In the Pennsylvania School for the Blind 33 per cent of the children admitted are blind from this cause. It is estimated that 20 to 30 per cent of all blindness in the United States is due to gonococcus infection. There are over 60,000 (?) blind in both eyes in this country. In Germany there occur 600 cases of blindness each year from this cause. In the institutions for the blind in Paris 46 per cent are so for one reason. Some claim that 60 per cent of those in institutions have lost their sight in this way. There is scarcely a physician in this country among the tens of thousands practicing who could not, were he to rend the veil of professional secrecy, point out instances in which the infection of father, transmitted to mother, has doomed the infant to a life of darkness.

Even in this disease, which a member of our legislature in drafting a bill to further safeguard marriage, omitted altogether, the State pays no small penalty. It is probable that it is one of the most frequent causes of divorce, although always covered up in the court records by such terms as "cruelty" and "non-support." The divorce evil, for such it is, disrupts homes and separates families to such an extent that it has not only become a sociological problem, but a problem for the Church and State. It is estimated that each person blind from birth is equivalent to a tax of $10,000 upon the people.

I have stated these facts somewhat in detail. They represent the conditions as observed by the best writers in the world. You fathers who have sons and daughters about to grow into young manhood and womanhood ought to know these things to protect them. You young men who hope to marry and become the heads of families ought to know them for the protection of the woman you hope to call wife.

In view of these facts, which are in no whit exaggerated, neither the young man nor the young woman is safe. Absalom died in battle, the victim of the vicissitudes of war. The victim of the venereal diseases lives on suffering worse than the tortures of the damned, marked of men, and wishing a thousand times that he might die. Do you think the conspiracy of silence enough to insure the safety of the young? Do you think that a feeling of false modesty should stand in the way of proper instruction? Do you not consider it your duty to show your child where the path of rectitude lies, and whither the path through prostitution leads? Does not the paternal instinct tell you it is better to instruct the confiding youth in these matters than to chide him when he has gone astray? The chiding is useless. He must pay the penalty.

With this condition of the social evil accepted as true, what is the solution of the problem? In my opinion suitable instruction will solve it in a generation or two, and this will need no sensational attachments. The parent is the natural guardian of the child, and he should be its natural instructor. This instruction should be given early, and later may be supplemented by the State. Orderly instruction through the proper channels will accomplish much. Much of the evil in the world is permitted evil. The parent who, either through ignorance or neglect, permits his son to fall by the wayside, is an enemy to society. The physician who, coming into the very closest relationship to the family, feels that his duty ends with the prescription counter, is an enemy to society. The minister who unites in marriage persons whom he knows to be mentally and morally unfit, is an enemy to society. The official who sees the evil in our midst and remains blind, is an enemy to society. Each and every one of these conspire to permit the gravest of crime under the guise of marriage. When this young man asks for your daughter's hand in marriage, will you require him to give proof of his freedom from venereal infection, or will you be content to know his income? When this young man asks your daughter to become his wife, has she not the right to require of him the same standard of purity that he demands in her? When she joins her life with his at the altar, has she no right to know that it is not the doorway to the divorce court, the operating room, the asylum or a life of invalidism?

I put these questions to you as a man having an interest in you. I put these questions to you as a husband, knowing what the wife does and has a right to expect of you. I put these questions to you as a father, knowing, and I hope appreciating, the rights of the child. I put these questions to you as a citizen knowing what the State must bear. Lastly, I put these questions to you as a physician knowing the dire consequences of the conspiracy of silence that permits the young man who wastes his life in the brothel to carry down in his fall those whom he should cherish.

Let us as men fight this battle to redeem manhood from its revolting servitude to vice and to protect and ennoble womanhood for the state of society grows better or worse with its estimate of woman.

---

PRECAUTIONS.—Ryan: For whoi 're puttin' up a fince, Doyle, afther all th' years ye've lived widout it?

Doyle: Well, th' fact is, Barney, th' docthor's bin at us t' take precautions against thim microbes ye've heard of.—*Tit-Bits.*

---

Small epigastric herniae, easily overlooked, are often the cause of pains in this region simulating those of stomach ulcer, gall-stones, etc. Some of these, however, consist only of properitoneal fat without any sac. The treatment of these latter by operation will not always relieve the pains for which a deeper source must, indeed, be sought.—*American Journal of Surgery.*

